# Beyond the Western front: targeted proteomics and organelle abundance profiling

**DOI:** 10.3389/fpls.2015.00301

**Published:** 2015-05-05

**Authors:** Harriet T. Parsons, Joshua L. Heazlewood

**Affiliations:** ^1^Section for Plant Glycobiology, Department of Plant and Environmental Sciences, University of Copenhagen, Frederiksberg, Denmark; ^2^Joint BioEnergy Institute, Physical Biosciences Division, Lawrence Berkeley National Laboratory, Berkeley, CA, USA; ^3^The Australian Research Council Centre of Excellence in Plant Cell Walls, School of BioSciences, The University of Melbourne, Melbourne, VIC, Australia

**Keywords:** multiple reaction monitoring (MRM), organelle abundance, immunoblotting, *Arabidopsis*, quantitative proteomics, proteomics

## Abstract

The application of westerns or immunoblotting techniques for assessing the composition, dynamics, and purity of protein extracts from plant material has become common practice. While the approach is reproducible, can be readily applied and is generally considered robust, the field of plant science suffers from a lack of antibody variety against plant proteins. The development of approaches that employ mass spectrometry to enable both relative and absolute quantification of many hundreds of proteins in a single sample from a single analysis provides a mechanism to overcome the expensive impediment in having to develop antibodies in plant science. We consider it an opportune moment to consider and better develop the adoption of multiple reaction monitoring (MRM)-based analyses in plant biochemistry.

Higher eukaryotic genomes encode tens of thousands of genes and after considering splice variants and post-translational modifications, likely produce hundreds of thousands of distinct protein products. In eukaryotic cells, proteins are found distributed amongst membrane bound organelles that undertake a multitude of specialized functions and often partition metabolic pathways. Understanding the functional roles of these organelles has given us a comprehensive overview of plant physiology, on to which the complex details of the dynamic regulation of plant-environment interactions can be mapped.

Subcellular fractionation and enrichment by density centrifugation has played a central role in elucidating the functional roles of subcellular compartments. The main biochemical processes were described years before the advent of electrophoretic transfer of proteins on to membranes or the use of antibodies to probe homogenates (Packer et al., 1970; Towbin et al., 1979; Burnette, 1981). Purity was typically assessed by a combination of electron microscopy and enzyme assays (Stocking, 1959; Douce et al., 1977; Mettler and Leonard, 1979) or, in some cases, radiolabeling (Galbraith and Northcote, 1977). Above a certain threshold of purity, maintenance of structural integrity and enzyme activity was the most important prerequisite during the fractionation process. However, for compartments that were less easily enriched than discrete organelles like the plastid or mitochondrion, assessment of contamination levels became more pressing and researchers turned toward immunoblotting, as well as enzyme assays and microscopy (Norman et al., 1986; Hahn et al., 1987; Meyer et al., 1988).

The advent of modern mass spectrometry and proteomics meant that not only could the main biochemical reactions or constituents of a compartment be investigated but many potentially functionally associated proteins could be identified, making organelle proteomics a valuable tool for reducing the complexity of the eukaryotic cell. The likelihood that a protein is correctly assigned to a location is either a function of the purity of the subcellular isolate (for review, see Millar and Taylor, 2014) or of the migration profile of an organelle on a continuous gradient, relative to other subcellular compartments (Dunkley et al., 2006; Nikolovski et al., 2012; Groen et al., 2014).This meant that accurate estimation of the organelle composition of samples became a critical question in this field as well as for biochemical analyses (Aronsson and Jarvis, 2002; Eubel et al., 2007; Parsons et al., 2012; Millar and Taylor, 2014). Although enzyme assays have proven useful in some contexts, as a general method for assessing organelle purity they are not suitable; maintenance of enzyme activity cannot always be assumed and, as reliable assays do not exists for all compartments, not all contaminants can be excluded. Purity is assayed most directly by electron microscopy, although as membranous vesicles can be difficult to distinguish, it cannot always provide a reliable answer. Furthermore, it is dependent on a considerable technical investment and knowledge that is not always possible for many research groups.

Immunoblotting provides a better means by which to address this question, but the qualitative nature of the signal detection makes it a poor choice for accurately assessing the proportional enrichment of a compartment. The availability of antibodies is not evenly distributed across the subcellular compartments in plants with some only being represented by one or two antibodies. Using multiple antibodies as representative markers for an organelle is an important control in situations where purity is paramount to the confidence placed in newly assigned proteins. Using publically available *Arabidopsis* proteomics data and spectral counting, it has been possible to estimate these confidence levels (Reumann et al., 2009; Parsons et al., 2012). However, quantification during fractionation nevertheless remains a limiting factor in this process.

The recent development of protein quantification methods by targeted mass spectrometry has revived discussions regarding the most efficient methods for the quantification of a protein in a sample (Lehmann et al., 2008; Aebersold et al., 2013). Targeted proteomics techniques aim to detect and determine the quantity of a limited set of predefined peptides in a complex mixture of peptides following enzymatic digestion of a protein samples by, e.g., trypsin. This is in contrast to data-dependent acquisition (commonly referred to as “shotgun proteomics”) where the aim is to identify as many peptides, and therefore proteins, in a sample as possible. This, however, introduces a certain element of randomness into peptide detection, particularly for lower-abundance peptides and so makes for poor protein quantitation. In multiple reaction monitoring (MRM), or selected reaction monitoring (SRM), a triple quadrupole mass spectrometer is used to select a precursor ion and its resultant product ion(s) after fragmentation (Kondrat et al., 1978). Selection of the parent ion occurs in the first mass analyzing quadrupole (Q1), which is set to a narrow mass window according to the masses of the ion(s) of interest. Collision induced disassociation in the second quadrupole (q2) results in fragmentation of the parent ion in to product ions which are detected in the third quadrupole (Q3) which, again, is set to an appropriately narrow mass window. By focussing machine time on a defined number of peptides, and by requiring both the parent and product ion to be detected, this technique is sufficiently sensitive and the background signal sufficiently low, that quantitation is possible for both high and moderately low-abundance peptides within the same complex starting mixture in a way that cannot be achieved using shotgun proteomics. The approach has been developed for proteomic studies, as demand for quantitative workflows has increased (Barnidge et al., 2003; Picotti et al., 2010; Maiolica et al., 2012). In recent years advocates have posited the technique as a superior alternative to immunoblotting (Maiolica et al., 2012; Aebersold et al., 2013; Picotti et al., 2013). Indeed, the application of MRM at the individual protein and protein isoform level has proved its ability to detect and quantify proteins against which raising antibodies would have been difficult (Zulak et al., 2009; Taylor et al., 2014). In *Arabidopsis* (Lehmann et al., 2008) and *Chlamydomonas* (Recuenco-Munoz et al., 2015), spiking samples with stable isotope-labeled versions of peptide targets has allowed absolute quantitation of proteins, referred to as a mass western as the results resemble the theoretical output of quantitative immunoblot but done using mass spectrometry.

Given the history of using approaches like westerns and enzyme assays to assess organelle contributions in a sample, the MRM technique could be extended from the individual protein to the compartment level by designing suites of peptide transitions covering marker proteins for multiple subcellular compartments. This would be akin to undertaking multiple immunoblots with suites of antibodies against major plant cellular compartments, like those currently available commercially (e.g., Agrisera AB) and would quickly and easily enable the estimation of the subcellular composition of a given sample. This perspective seeks to explore MRM as an alternative to immunoblotting for assessing the relative abundance of organelles in plant homogenates.

Unlike many targeted approaches using mass spectrometry where protein abundance is assayed in the context of a response, this survey describes the relative abundance of marker proteins between compartments in the same sample, without reference to their function. Several marker proteins and representative peptides per compartment were selected to ensure the overall signal would be representative of the compartment as a whole. Once adequately developed with a collection of reliable transitions that had been assessed for parameters such as limits of detection, limits of quantitation, matrix effects, ion suppression and linearity, the adoption of this technique could greatly benefit the plant community. The ability to assess both the contamination levels of an organelle preparation and track organelle migration during centrifugation would be incredibly useful, but it is imagined that it could also provide means for the rapid monitoring of changes in organelle populations (Yan et al., 2005; Castillo et al., 2008). Consequently, we sought to highlight the potential of the approach by developing an initial set of transitions for specific organelle marker proteins to assess the potential of this approach.

An organelle abundance profile was generated for the reference plant *Arabidopsis* by selecting and analyzing candidate MRM peptide transitions for a number of organelle marker proteins (Figure 1). Only proteins repeatedly localizing to a subcellular compartment (Tanz et al., 2013) and generating non-redundant peptides were selected as markers. As far as was possible, selected proteins were functionally unrelated, not co-expressed and within the top 40 most expressed transcripts for an organelle or compartment. This last point was important for comparisons between compartments. For this proof-of-concept study a minimum of three marker proteins per compartment was applied (with the exception of the vacuole); in some instances up to five were employed (e.g., for the PM) when available MRM transitions were readily identified (Table 1). A ribosomal category was included with the 10 major subcellular categories (Table 1) as these can be an appreciable source of sample contamination in subcellular proteomics. Using *in vitro* synthesis techniques (Brownridge et al., 2011), we have thus far validated the identity (retention time and fragment ions) of at least one peptide per subcellular compartment, i.e., 25 of the 72 peptides.

**FIGURE 1:**
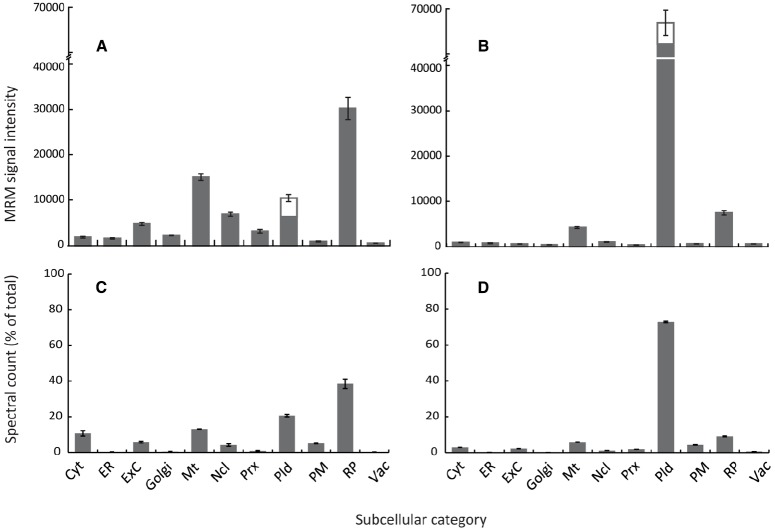
***Arabidopsis* organelle profiling by MRM and shotgun proteomics.** Estimation of relative abundance of cellular compartments from total protein extracts of 7-day old cell suspension cultures **(A,C)** or 4-week rosettes **(B,D)**. MRM assays **(A,B)** were performed using two to five marker proteins per compartment, except the plastid where six marker proteins were used, including both the light-harvesting complex candidates (three proteins) and non-light harvesting complex candidates (three proteins). Error bars show standard error for *n* = 3 biological replicates. Spectral counts **(C,D)** were obtained from data-dependent LC-MS/MS analysis of total protein extracts with around 1,500 proteins identified for each tissue using Mascot (*p* < 0.05 Ions score). Organelle marker proteins lists were generated from the SUBcellular *Arabidopsis* database (Tanz et al., 2013). Spectra for all proteins matching each organelle/subcompartment were summed and expressed as a percentage of the total number of identified proteins. Cyt, cytosol; ER, endoplasmic reticulum; ExC, extracellular; Mt, mitochondria; Ncl, nucleus; Prx, peroxisome; Pld, plastid; PM, plasma membrane; RP, ribosomal proteins; Vac, vacuole.

**TABLE 1:** **Summary of representative marker proteins and peptides used for detection of subcellular compartments by MRM**.

**Subcellular category**	**Marker proteins**	**Marker peptides**	**Peptides verified**
Cytosol	4	7	2
ER	4	6	1
Extracellular	3	5	1
Golgi	3	6	2
Mitochondria	4	5	1
Nucleus	3	5	2
Peroxisome	3	5	2
Plastid (LHC^1^)	3	5	2
Plastid (non-LHC^1^)	4	8	4
PM	5	9	3
Ribosomes	3	7	2
Vacuole	2	2	1

^1^LHC, light harvesting complex.

Typical differences in organelle abundance detected using this MRM method are demonstrated in two very different but popular experimental systems; heterotrophic *Arabidopsis* cell-suspension culture (Figure 1A) and 4-week old *Arabidopsis* rosettes (Figure 1B). As growth conditions varied dramatically between systems, particularly with respect to light and carbon source, both light-harvesting complex and non-light harvesting complex plastid markers were included. These MRM profiles of subcellular compartments were then compared to profiles generated by spectral counts of several 100 compartment marker proteins from data-dependent analyses of total protein extracts (Figure 1; Table 1). Although data-dependent acquisition approaches are known to favor medium/high abundance proteins (Wienkoop and Weckwerth, 2006; Ahn et al., 2007), since relatively abundant proteins had been selected as organelle markers for MRM such a comparison was considered meaningful. Both MRM and data-dependent analyses produced similar organelle profiles for each system (Figure 1), showing that using MRM to estimate the relative abundance of subcellular compartments is conceptually valid. Changes in relative abundance were detected in all subcellular categories, demonstrating the quantitative capacity and sensitivity of MRM. As expected given the physiological differences between the two systems, plastids were much less abundant, and mitochondria more so, in cell cultures compared to rosettes (Figures 1A,B). Ribosomal proteins and lower-abundance organelles such as the Golgi and peroxisome appeared more abundant in cell cultures (Figure 1A), as expected for cytoplasmic-dense, rapidly-dividing, undifferentiated cells grown in a relatively high-oxygen environment.

Some differences between spectral counting and MRM were observed. Lower-abundance organelles such as the Golgi, peroxisome and ER appeared lower when estimated by spectral counting (compare Figures 1A,C). The ratio of plastidic proteins to proteins from other compartments also appears lower in the MRM results compared to standard spectral counting approaches (Figures 1B,D). Detection is biased against very small, low abundance, or hydrophobic proteins using data-dependent acquisitions, particularly in complex samples, whilst heavily post-translationally modified proteins may never be detected. Undoubtedly, this will affect compartments disproportionately, potentially leading to misrepresentation using techniques such as spectral counting, which could explain these discrepancies between results such as over-representation of the plastid in photosynthetic tissues. This analysis demonstrates a proof of concept for this application of MRM in determining relative organelle abundance, and shows how it could potentially lead to a more accurate estimation of organelle abundance when compared to immunoblotting, enzyme assays, and other mass spectrometry techniques such as spectral counting. However, these results do also point to some potential drawbacks of this technique in its current format.

This technique relies on the assumption that changes in abundance of an entire subcellular compartment can be represented by a small number of proteins. Therefore, this makes the appropriate selection of proteins a critical consideration when designing suites of transitions for detecting subcellular compartments. Tissue- or environment specific changes in gene expression can be largely avoided by consulting publically available microarray data; however how this compares to proteins levels is harder to predict. The disproportionate decrease in light-harvesting complex proteins (Figures 1A,B) may reflect environmental influence on protein expression. However, the similarities between Figures 1A,C and Figures 1B,D suggest that by applying stringent criteria during MRM marker selection, such effects may be minimized. For example, had markers been selected entirely from non-light harvesting complex proteins, our results would have looked very different.

Through this proof-of-concept study, which paves the way for an in-depth analysis of the applicability of MRM to estimating the subcellular composition of samples, we demonstrate that relative quantitation can provide a sufficient overview. One established such approaches could enable profiling of organelles abundance between treatments or as a means to assess the purity of a subcellular fraction. Although this technique does not offer absolute peptide quantitation, as can be achieved by using labeled peptides (Lehmann et al., 2008), this technique likely represents an appreciable increase in accuracy compared to immunoblotting and would already be applicable to a well-annotated species such as *Arabidopsis*. By avoiding isotope-labeled peptides used for absolute quantification, e.g., AQUA (Gerber et al., 2003), which requires additional levels of cost, we aim to increase accessibility of this technique. The triple quadrupole mass spectrometers required are relatively affordable, further increasing the potential popularity of MRM for such approaches. Future design of peptide transitions will be facilitated by the MRMaid and *Arabidopsis* Proteotypic Predictor resources (Fan et al., 2012; Taylor et al., 2014) and validation of transitions facilitated by the development of synthetic peptides libraries (Picotti et al., 2010) and the emergence of techniques such as QconCAT (Beynon et al., 2005; Brownridge et al., 2011). The investment required for quality antibody development will likely prevent production keeping pace with the number of emerging organisms of research interest. Genomic annotation of several more plant species is approaching the level of completion required for non-redundant peptide selection (Goodstein et al., 2012), whilst ever accumulating data in PRIDE (Vizcaino et al., 2013) will facilitate the design of marker peptides for other species, meaning that MRM-based techniques will have a sizable impact on plant cellular biology in the coming years.

## Conflict of Interest Statement

The authors declare that the research was conducted in the absence of any commercial or financial relationships that could be construed as a potential conflict of interest.
